# Hemolysin function of *Listeria* is related to biofilm formation: transcriptomics analysis

**DOI:** 10.1186/s13567-022-01124-y

**Published:** 2022-12-31

**Authors:** Ruidan Li, Qian Liang, Sicheng Tian, Yunwen Zhang, Sijing Liu, Qian Ou, Zhaobin Chen, Chuan Wang

**Affiliations:** 1 Department of Public Health Laboratory Sciences, West China School of Public Health and West China Fourth Hospital, Sichuan University, Chengdu, 610061 China; 2 Shen Zhen Biomed Alliance Biotech Group Co., Ltd, Shenzhen, 518057 China

**Keywords:** Transcriptomics, *Listeria* hemolysin, biofilm, virulence factors

## Abstract

Listeriolysin O (LLO) is the main virulence protein of *Listeria monocytogenes* (LM), that helps LM escape lysosomes. We previously found that the cellular immune response elicited by *L.ivanovii* (LI) is weaker than that elicited by LM. We speculated that this may be related to the function of ivanolysin O (ILO). Here, we constructed hemolysin gene deletion strain, LIΔ*ilo*, and a modified strain, LIΔ*ilo*::*hly*, in which *ilo* was replaced by *hly*. Prokaryotic transcriptome sequencing was performed on LI, LIΔ*ilo*, and LIΔ*ilo*::*hly*. Transcriptome differences between the three strains were compared, and genes and pathways with significant differences between the three strains were analyzed. Prokaryotic transcriptome sequencing results revealed the relationship of *ilo* to the ribosome, quorum sensing, and phosphotransferase system (PTS) pathways, etc. LIΔ*ilo* exhibited attenuated biofilm formation ability compared to LI. Biofilm formation was significantly recovered or even increased after replenishing *hly*. After knocking out *ilo*, the relative expression levels of some virulence genes, including *sigB*, *prfA*, *actA*, *smcL*, and *virR*, were up-regulated compared to LI. After replenishing *hly*, these genes were down-regulated compared to LIΔ*ilo.* The trend and degree of such variation were not completely consistent when cultured in media containing only monosaccharides or disaccharides. The results confirmed that hemolysin is related to some important biological properties of *Listeria*, including biofilm formation and virulence gene expression levels. This is the first comprehensive study on ILO function at the transcriptomic level and the first evidence of a relationship between *Listeria* hemolysin and biofilm formation.

**Supplementary Information:**

The online version contains supplementary material available at 10.1186/s13567-022-01124-y.

## Introduction

*Listeria monocytogenes* (LM) is a gram-positive, non-budding, short bacterium. It is widely distributed in natural environments, including sewage and soil [1]. It was first discovered in 1926 during an outbreak in rabbits and guinea pigs [2]. LM is also an important food-borne pathogen responsible of the human listeriosis and capable of persisting in food industry by forming biofilms [3, 4]. LM is also an intracellular parasite that can induce a cellular immune response in the host [5]. The bacterium was initially used as a model organism for studying the mechanisms of cellular immunity [6]. Because LM can induce a potent cellular immune response, its value as a vaccine carrier has been explored. In one study involving an attenuated LM strain, knocking out of the *dal* and *dat* genes and inserting of the human *CD24* gene led to the regression of the subcutaneously inoculated Hepa1-6-CD24 cell-derived tumor and increased tumor-free survival in mice [7].

The main virulence factor of LM is listeriolysin O (LLO), which is encoded by *hly* [8]. The gene is located on the LM-first pathogenicity island (LIPI-1) [9]. LLO is associated with the unique intracellular lifestyle of LM, and it is important for the ability of LM to escape from intracellular phagocytic vesicles to induce cellular immunity [10]. Inactivation of LLO results in the loss of hemolytic activity, blockage of phagosomal escape, and reduced virulence in mice [11–13]. In one study, researchers observed the escape ability of *hly-*inactivated-LM from phagocytic vesicles by scanning electron microscopy (SEM) [14]. The findings implicated LLO as a virulence factor necessary for the bacteria to escape from internalized vesicles.

*Listeria ivanovii* (LI) is the only pathogenic bacterium of the genus *Listeria* other than LM. LI almost always only infects ruminants and human infections are rare [15]. It was first isolated from a lamb with congenital listeriosis in Bulgaria in 1955 [16]. LI has properties similar to LM, such as intracellular parasitism and direct intercellular transmission, and can enter and proliferate in antigen-presenting cells, such as macrophages [17]. Our group has ever constructed several recombinant LI vectored vaccines and demonstrated that they can all induce antigen-specific CD4^+^ and CD8^+^ T cell immune response [18]. However, we also found that the cellular immune response induced by LI vectored vaccine was weaker than that of corresponding LM vectored vaccine, showing the immunogenicity of LI is not as strong as LM.

The intracellular parasitism by LM and its ability to induce cellular immune responses are primarily related to LLO. Ivanolysin O (ILO), which is encoded by *ilo*, can replace LLO in vitro [8], indicating that ILO has similar functions to LLO. However, in most studies, ILO function was indirectly analyzed by replacing LLO with ILO. Direct examination of ILO function have not yet been reported. In one study, researchers constructed a recombinant strain, LMΔ*hly*::*ilo*, by replacing *hly* with *ilo* in the bacterial genome [17]. The recombinant LM strain could proliferate in the liver of mice, but not in the spleen, suggesting that the functions of ILO and LLO were not completely identical. ILO-mediated bacterial proliferation in the spleen was weaker than that in LLO, and the ability of ILO to activate the immune response was weaker than that of LLO. Another study [17] confirmed that ILO has a weaker ability than LLO to assist bacteria in escaping phagocytic vesicles into host cells. Based on these findings, we speculated that the reason for the weaker immunogenicity of LI compared with LM may be related to the function of ILO. Compared to the more thorough studies on LM and LLO, there are relatively few studies on LI and ILO as of July 2022, with only 10 results in PubMed using the search term “ivanolysin O”.

Thoroughly exploring the function of ILO will enrich the knowledge of ILO. This research aims to explain the function of ILO to a certain deeply degree, and provide a theoretical basis for optimizing the immunogenicity of LI, which is valuable for the application of LI vaccine carriers. In the present study, we constructed a hemolysin gene deletion LI strain, LIΔ*ilo*, and a hemolysin gene-modified LI strain, LIΔ*ilo*::*hly*, in which *ilo* was replaced by *hly*. Prokaryotic transcriptome sequencing was performed and genes and pathways with significant differences between the three strains were analyzed.

## Materials and methods

### Bacteria

LM 10403s and LI PAM55 were kindly provided by Dr Hao Shen (Department of Microbiology, Perelman School of Medicine, University of Pennsylvania). Plasmids pCW619, pCW620 (which harbors *lacZ*), and pCW621 (which harbors *hly*) were constructed by our group. Plasmid pCW620 was electroporated into LI and the strain LIΔ*ilo*::*lacZ* was constructed by homologous recombination [19]. Plasmids pCW619 and pCW621 were electroporated into LIΔ*ilo*::*lacZ* to construct strains LIΔ*ilo* and LIΔ*ilo*::*hly*, respectively. Schematic diagrams of the targeted plasmids pCW619, pCW620, and pCW621, and the recombinant strains are presented in Additional file 1.

### Bioinformatics analyses of LLO and ILO

Serial Cloner software was used to compare the nucleotide sequences of the LLO protein-coding gene *hly* (GenBank: DQ054589.1) and the ILO protein-coding gene *ilo* (GenBank: X60461.1). The translation and open reading frames were predicted using DNASTAR software and the Expasy Translate tool, and the amino acid sequences were compared using DNAMAN software. The pI, Mw, instability index, and aliphatic index were predicted using the ProtParam and Compute pI/Mw tools. Secondary structures were predicted using Predict Protein, SOPMA, and PSIPRED software. Tertiary structures were predicted by Swiss-Model and analyzed using the PDBsum Generate. Hydrophilicity and hydrophobicity were predicted using ProtScale. The transmembrane structures were predicted using TMHMM Server v. 2.0. The functional domains were predicted using CDD. Information concerning the database, bioinformatics analysis software, and websites are presented in Table 1.

**Table 1:** **Database and bioinformatics analysis software and websites**

Software	Websites
NCBI	https://www.ncbi.nlm.nih.gov/
ExPASy	https://web.expasy.org/compute_pi/
ProtParam tool	https://web.expasy.org/protparam/
Compute pI/Mw tool	https://web.expasy.org/compute_pi/
Predict protein	https://predictprotein.org/
SOPMA	https://npsa-prabi.ibcp.fr/cgi-bin/npsa_automat.pl?page=npsa_sopma.html
PSIPRED	http://bioinf.cs.ucl.ac.uk/psipred/
Swiss-model	https://swissmodel.expasy.org/interactive
PDBsum generate	https://www.ebi.ac.uk/thornton-srv/databases/pdbsum/Generate.html
ProtScale	https://web.expasy.org/protscale/
TMHMM server v. 2.0	http://www.cbs.dtu.dk/services/TMHMM/
CDD	https://www.ncbi.nlm.nih.gov/Structure/cdd/wrpsb.cgi

### **Prokaryotic transcriptomic sequencing of LI, LIΔ*ilo***, **and LIΔ*ilo***::***hly*strains**

Total RNA was isolated using the TRIzol reagent (Invitrogen Life Technologies, USA). The sequencing library was sequenced on a NextSeq 500 platform (Illumina, USA) in Shanghai Personalbio Technology Co., Ltd. DEGs obtained by transcriptome sequencing analysis were verified using RT-qPCR. The RT-qPCR reaction system and conditions were according to the manufacturer’s instructions (SsoFast EvaGreen Supermix, Bio-Rad, China). The 16s rRNA universal primer was used as the internal reference, and the relative transcription level of each gene was calculated using the 2^-ΔΔCt^ method.

Two-way cluster analysis of the union of all differentially expressed genes (DEGs) in all groups and samples was performed. Cluster analysis was performed based on the transcription level of the same gene in different samples and the expression patterns of different genes in the same sample. The Euclidean method was used to calculate distance and the hierarchical clustering method (complete linkage) was used for clustering.

### Phenotypic analysis of differential pathways

#### Quorum sensing pathway—biofilm formation

Biofilms were cultured as previously described [18], with slight modifications. One milliliter of BHI broth culture (Landbridge, China) containing 1 × 10^7^ CFU/mL of LM, LI, LIΔ*ilo*, and LIΔ*ilo*::*hly* was added to each well of a 24-well PVC plate (Corning, USA) and cultured at 37 °C for 1, 2, 3, and 4 days. The culture medium was removed, and the wells were washed twice with PBS and air-dried to obtain biofilms. Crystal violet staining was performed immediately or after adding 0.1 mg/mL proteinase K (Solarbio, China) at 37 °C for 3 h. Crystal violet staining was performed as follows. Methanol (1 mL) was added to each well for fixation for 15 min. The methanol was removed and 1 mL of 0.1% crystal violet (Solarbio, China) was added for 10 min. Crystal violet was removed, each well was washed three times with PBS, dried, and resuspended in 1 mL of 33% acetic acid. The supernatant was transferred to a new plate and the optical density at 595 nm was measured using a microplate reader (Thermo Fisher Scientific, USA).

In addition to crystal violet staining, viable bacteria in the biofilm were determined. One milliliter of PBS was added to each well, and ultrasonic treatment was performed using an Elmasonic P apparatus (Elma, Germany) operating at 37 kHz for 5 min. Twenty microliters of the suspension were added to a BHI (Landbridge, China) plate cultured at 37 °C for 48 h. Colonies were enumerated and the viable count was expressed as CFU.

One milliliter BHI broth culture (Landbridge, China) containing 1 × 10^7^ CFU/mL of LM, LI, LIΔ*ilo*, and LIΔ*ilo*::*hly* were added to each well of a 24-well PVC plate (Corning, USA), with a sterile slide placed in each well. After incubation at 37 °C for 3 days, the slides were removed and washed twice with distilled deionized water.

The slides were placed in 2.5% glutaraldehyde solution for 12 h, dehydrated with successive concentration gradients of ethanol solution (30%, 50%, 70%, 90%, and 100%), dehydrated for 10 min, and then removed for critical point drying. The slides were gold sprayed and then observed with a SEM (Inspect F, FEI, Netherlands).

Two hundred microliters of the cell membrane staining working solution (LIVE/DEAD™ BacLight™ Bacterial Viability Kit; Invitrogen, USA) were added to the slides. Live bacteria were labeled with cyto9 dye and dead bacteria were labeled with PI dye. The slides were incubated at 37 °C for 15 min in the dark, washed three times with distilled deionized water, removed, and placed on glass slides. After mounting, the slides were observed by LSCM using a model A1R^+^ microscope (Nikon, Japan). NIS Elements and ImageJ software were used to image and calculate the thickness and fluorescence intensity of the biofilm.

#### PTS pathway—virulence gene transcription

LI, LIΔ*ilo*, and LIΔ*ilo*::*hly* were inoculated into a TSB medium (aladdin, China) with 0.5% glucose or 0.5% cellobiose as the sole carbon source and incubated at 37 °C. The bacteria were cultured to an optical density at 600 nm of 0.4–0.5. Then, bacterial RNA was extracted using an RNAprep pure Cell/Bacteria Kit (Tigen, China). RNA was reverse transcribed into cDNA using TransScript One-Step gDNA Removal and cDNA Synthesis SuperMix (Transgen, China).

### Statistical analyses

Data were processed using SPSS 21.0 (IBM, USA). Those with a normal distribution are expressed as the mean ± standard deviation. One-way ANOVA was used for parametric tests, and the LSD test was used for pairwise comparisons between groups. *P* < 0.05 indicated that the difference was statistically significant.

## Results

### Bioinformatics analyses of LLO and ILO

**Figure 1:**
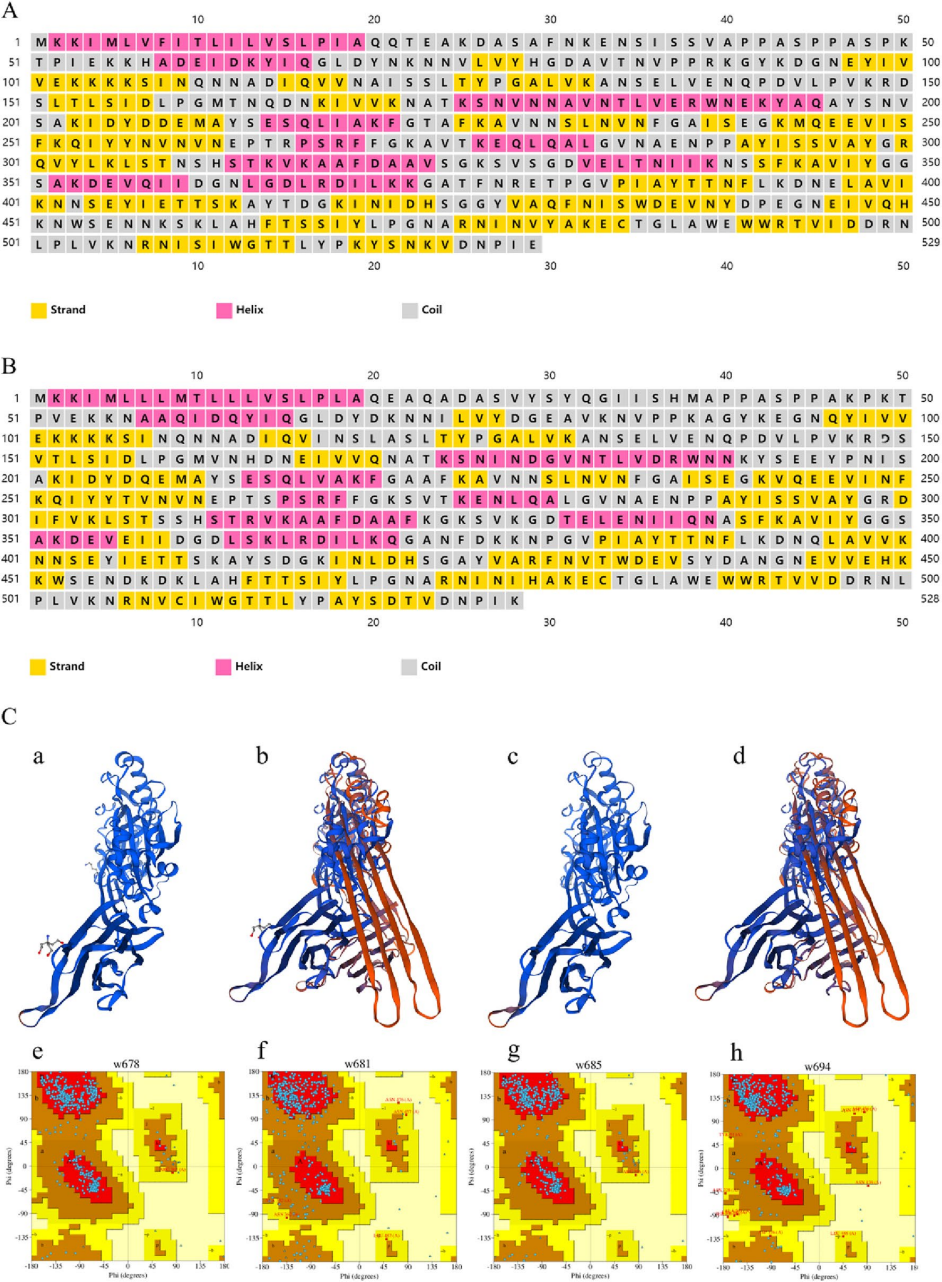
**Bioinformatics analysis of LLO and ILO proteins.** Secondary structures of LLO **A** and ILO **B** predicted by PSIPRED software; The tertiary structure of LLO (panels a, b) and ILO (panels c, d) predicted by Swiss-Model software **C** and Ramachandran plot of LLO Swiss-Models (panels e, f) and ILO Swiss-Models (g, h) estimated by PDBsum Generate software. In Ramachandran plot, red regions indicate the most favoured region, brown regions indicate the additional allowed region, yellow regions indicate the generously allowed region and light yellow regions indicate the disallowed region. The blue dots indicate the individual amino acids that make up the protein.

The sequence homology between *hly* and *ilo* was 76.64% and the translated amino acid sequence homology was 78.03%. The isoelectric point (pI), molecular weight (Mw), instability index, and aliphatic index of the LLO and ILO are listed in Table 2. Both LLO and ILO are stable. Asparagine is the most prevalent amino acid component (10.4%) (Additional files 2A, B). The secondary structures of LLO (Additional file 2C) and ILO (Additional file 2D) predicted by the PredictProtein software showed that both are mixed structural proteins. The secondary structures of LLO (Additional file 2E) and ILO (Additional file 2F) predicted by SOPMA software showed that in LLO, proportions of *α*-helix, extended chain, *β*-turn, and random coil were 25.90%, 24.57%, 6.99%, and 42.53%, respectively. The respective proportions in ILO were 26.14%, 24.62%, 6.25%, and 42.99%. The secondary structures of LLO (Figure 1A) and ILO (Figure 1B) predicted by the PSIPRED software revealed dominant random coils, extended chains, and *α*-helices, with random coils accounting for the highest proportion. Swiss-Model software predicted the tertiary structures of LLO and ILO (Figure 1C). Two LLO protein prediction models were obtained. Model a (Figure 1C, panel a) showed its reference template protein was 4cdb1.a (SWISS-MODEL Template Library), the model circumference of residual infrastructure was 39–526, the sequence similarity was 0.61, and the template coverage was 0.92. Model b (Figure 1C, panel b) showed its reference template protein was 5ly6.1.a (SWISS-MODEL Template Library), the model circumference of residual infrastructure was 58–527, the sequence similarity was 0.42, and the template coverage was 0.89. For the two ILO protein prediction models, model c (Figure 1C, panel c) showed its reference template protein was 4cdb1.A (SWISS-MODEL Template Library), the model circumference of residual infrastructure was 38–525, the sequence similarity was 0.56, and the template coverage was 0.92. Model d (Figure 1C, panel d) showed its reference template protein was 5ly6.1.a (SWISS-MODEL Template Library), the model circumference of residual infrastructure was 56–525, the sequence similarity was 0.43, and the template coverage was 0.89. The Ramachandran plot of each model (Figure 1D) confirmed that the prediction models were reasonable, especially Model a and c. Ramachandran plot (PDB ID: W678) (Figure 1D, panel a) corresponded to Model a showed that 100% of the amino acid residues were in the reasonable region, including 91.8% in the most favoured region, 8.0% in the additional allowed region, 0.2% in the generously allowed region. Raman diagram (PDB ID: W685) (Figure. 1D, panel c) corresponded to Model c showed that 100% of the amino acid residues were in the reasonable region, including 91.1% in the most favoured region, 8.7% in the additional allowed region, 0.2% in the generously allowed region. ProtScale software predicted the hydrophilicity and hydrophobicity of LLO (Additional file 2G) and ILO (Additional file 2H); both of which were hydrophilic. TMHMM Server v. 2.0 software predicted the transmembrane structures of LLO (Additional file 2I) and ILO (Additional file 2J); it showed none of them was predicted to be transmembrane protein, but more likely to be extracellular proteins. CDD software predicted the functional domains of LLO (Additional file 2K) and ILO (Additional file 2L). Both LLO and ILO are thiol-activated cytolysin family proteins (domain architecture ID: 13651282), and both contain two functional domains: thiol_cytolysin (pfam01289) and thiol_cytolys_C (pfam17440). The first one is a thiol-activated cytolysin and the latter is a thiol-activated cytolysin *β* sandwich domain.

**Table 2:** **Physicochemical properties of LLO and ILO**

Index	LLO	ILO
Gene sequence homology	76.64%	
Amino acid sequence homology	78.03%	
Number of amino acid residues	529	528
Number of amino acids with positive and negative charges	60, 59	58, 43
Number of polar and non-polar amino acids	168, 180	159, 184
pI	7.63	5.93
Mw	58646.00	58511.90
Instability index	34.25	29.39
Aliphatic index	85.14	86.23

### Transcriptomic sequencing and analysis

**Figure 2:**
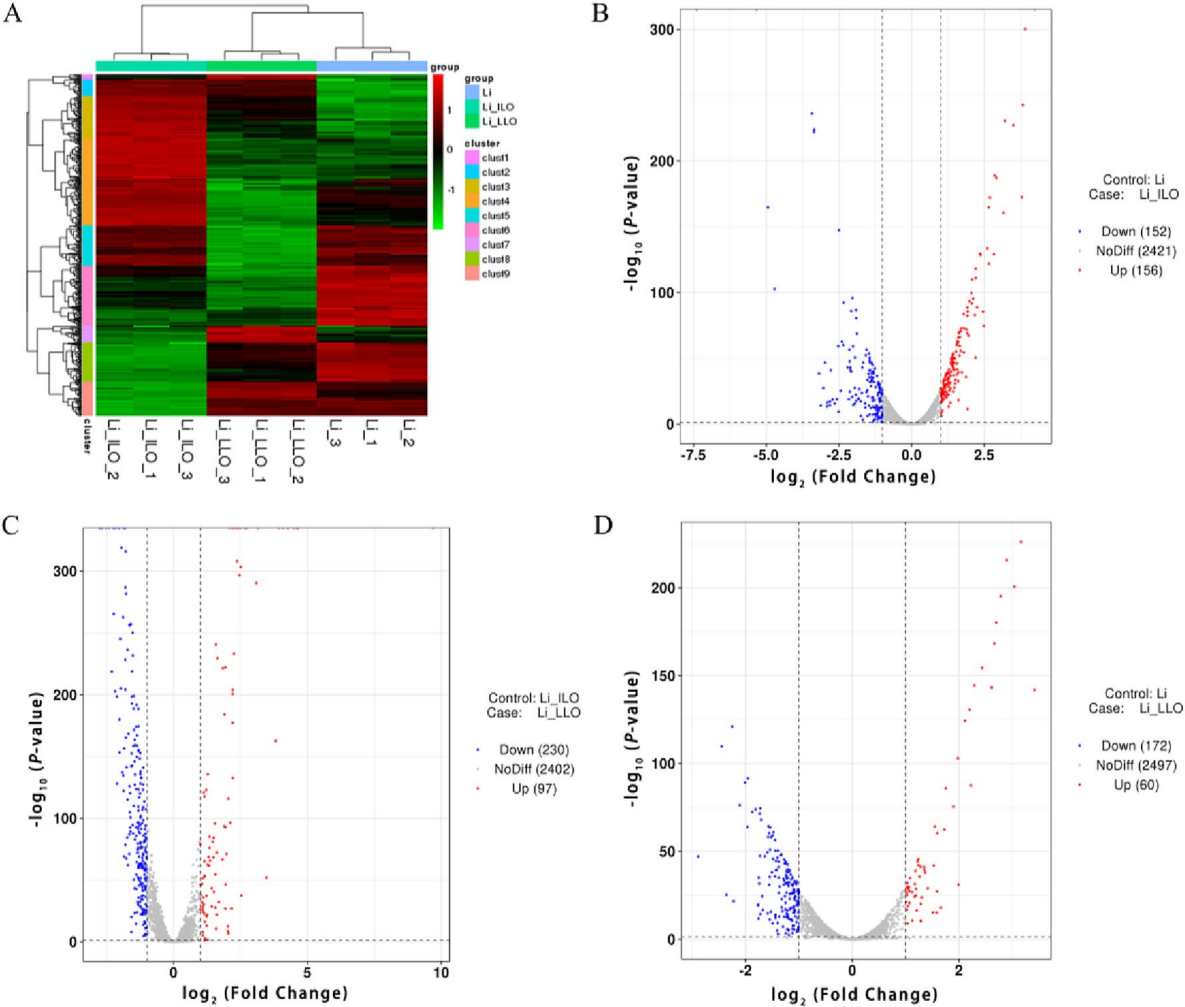
**Transcription level and expression difference analysis.** Cluster analysis: heat map showing statistical trends for differentially expressed genes in LI, LIΔ*ilo*, and LIΔ*ilo*::*hly*. **A** (genes are represented horizontally, each column represents a sample, red represents up-regulated genes, green represents down-regulated genes, and black represents non-differentially expressed genes); Detection of differentially expressed genes, LIΔ*ilo* vs. LI **B**, LIΔ*ilo*::*hly* vs. LIΔ*ilo* **C**, LIΔ*ilo*::*hly* vs. LI **D** (volcano plot: abscissa is log_2_ (Fold Change), ordinate is -log_10_ (*P*-value), the two vertical dotted lines in the figure are the 2-fold expression difference threshold; the horizontal dotted line is the *P*-value = 0.05 threshold, red dots indicate up-regulated genes, blue dots indicate down-regulated genes, and gray dots indicate non-significantly differentially expressed genes). *n =* 3.

The clustering result showed that genes were differentially expressed in LI, LIΔ*ilo*, and LIΔ*ilo*::*hly*. Most genes showed opposite trends among LI, LIΔ*ilo*, and LIΔ*ilo*::*hly* (Figure 2A). Volcano plots showed that LIΔ*ilo* had 156 up-regulated genes and 152 down-regulated genes compared to LI (Figure 2B); LIΔ*ilo*::*hly* had 97 up-regulated genes and 230 down-regulated genes compared to LIΔ*ilo* (Figure 2C); LIΔ*ilo*::*hly* had 60 up-regulated genes and 172 down-regulated genes compared with LI (Figure 2D).

**Figure 3:**
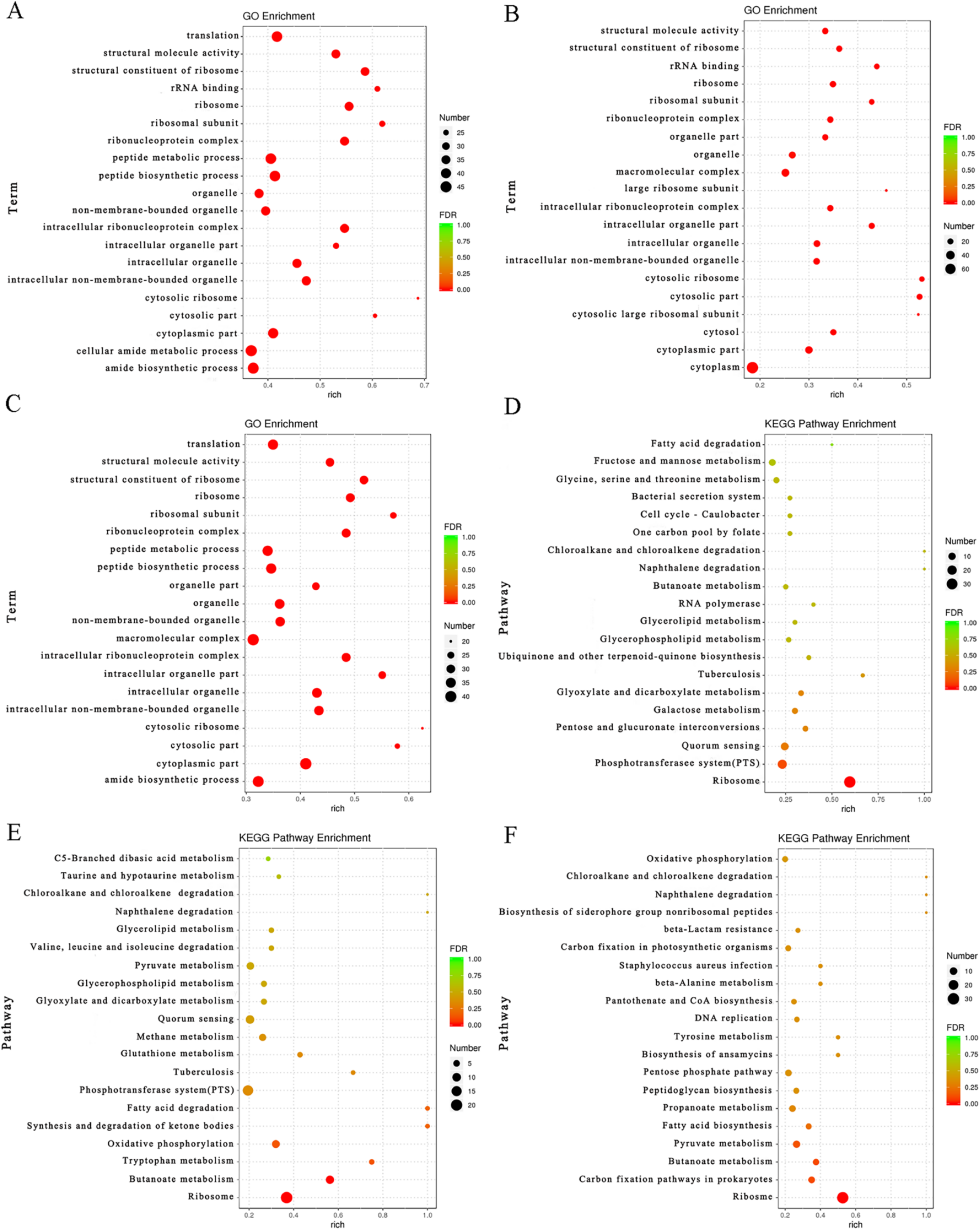
**Functional enrichment analysis of differentially expressed genes.** GO enrichment analysis bubble plot, LIΔ*ilo* vs. LI **A**, LIΔ*ilo*::*hly* vs. LIΔ*ilo* **B**, LIΔ*ilo*::*hly* vs. LI **C**. KEGG enrichment analysis bubble plot, LIΔ*ilo* vs. LI **D**, LIΔ*ilo*::*hly* vs. LIΔ*ilo* **E**, LIΔ*ilo*::*hly* vs. LI **F**; *n* = 3.

Gene ontology (GO) enrichment analysis showed that compared with LI, the top five items with significant in LIΔ*ilo* were structural constituent of ribosome, ribosome, intracellular ribonucleoprotein complex, ribonucleoprotein complex, and structural molecule activity (Figure 3A). Compared with LIΔ*ilo*, the top five items with significant in LIΔ*ilo*::*hly* were cytosolic part, cytosolic ribosome, intracellular organelle part, rRNA binding, and ribosomal subunit (Figure 3B). Compared with LI, the top five items with significant in LIΔ*ilo*::*hly* were cytoplasmic part, structural constituent of ribosome, ribosome, intracellular ribonucleoprotein complex, and ribonucleoprotein complex (Figure 3C).

Kyoto Encyclopedia of Genes and Genomes (KEGG) enrichment analysis showed that compared with LI, ribosome, phosphotransferase system (PTS), and quorum sensing were the most enriched pathways in LIΔ*ilo* (Figure 3D). Compared with LIΔ*ilo*, ribosome, PTS, and quorum sensing were the most significantly enriched pathways in LIΔ*ilo*::*hly* (Figure 3E). Compared with LI, ribosome and carbon fixation pathways in prokaryotes and butanoate metabolism were the most significantly enriched pathways in LIΔ*ilo*::*hly* (Figure 3F).

Based on the KEGG pathway enrichment analysis results, we selected the quorum sensing and PTS pathways that were related to bacterial virulence for further study. Compared with LI, the proteins expression levels of AgrA, AgrB, and AgrC were all down-regulated in LIΔ*ilo* (Additional file 3A). Compared with LIΔ*ilo*, the protein expression level of AgrB was up-regulated in LIΔ*ilo*::*hly* (Additional file 3B). In LIΔ*ilo*::*hly*, the proteins expression levels of AgrA, AgrB, and AgrC were recovered to a level comparable to that of LI (Additional file 3C).

**Figure 4:**
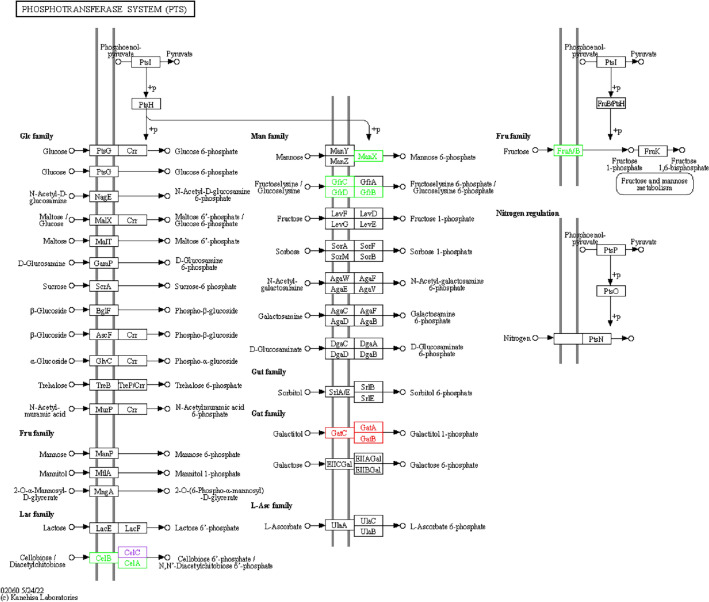
**KO analysis of metabolic pathways.** PTS pathway, LIΔ*ilo* vs. LI. Boxes represent proteins. Red nodes represent up-regulated proteins; green nodes represent down-regulated proteins; purple nodes represent up-regulated proteins compared to the reference genome LI used for transcriptome sequencing.

Compared with LI, the proteins expression levels of CelA, CelB, ManX, GfrB, GfrC, GfrD, and FruA/B were down-regulated in LIΔ*ilo*; the proteins expression levels of GatA, GatB, and GatC were up-regulated in LIΔ*ilo* (Figure 4).

**Figure 5:**
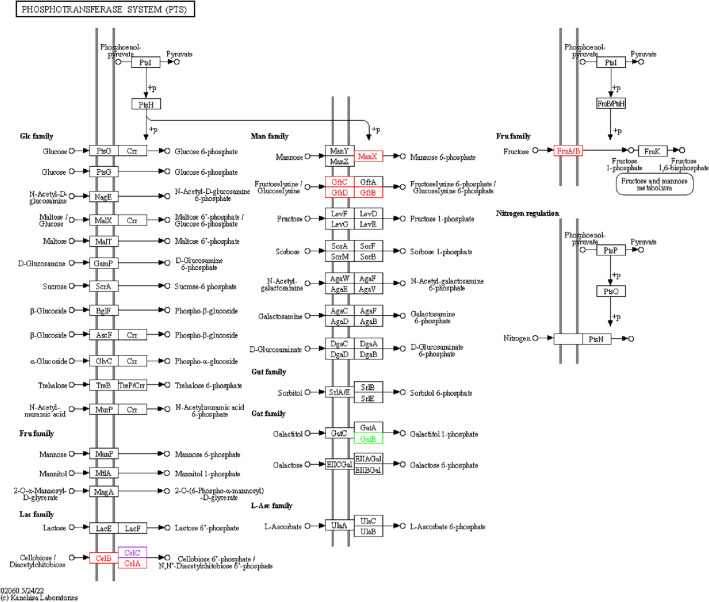
**
KO analysis of metabolic pathways.** PTS pathway, LIΔ*ilo*::*hly* vs. LIΔ*ilo*. Boxes represent proteins. Red nodes represent up-regulated proteins; green nodes represent down-regulated proteins; purple nodes represent up-regulated proteins compared to the reference genome LI used for transcriptome sequencing.

Compared with LIΔ*ilo*, the proteins expression levels of CelA, CelB, ManX, GfrB, GfrC, GfrD, and FruA/B were up-regulated in LIΔ*ilo*::*hly*; the protein expression level of GatB was down-regulated in LIΔ*ilo*::*hly* (Figure. 5).

**Figure 6:**
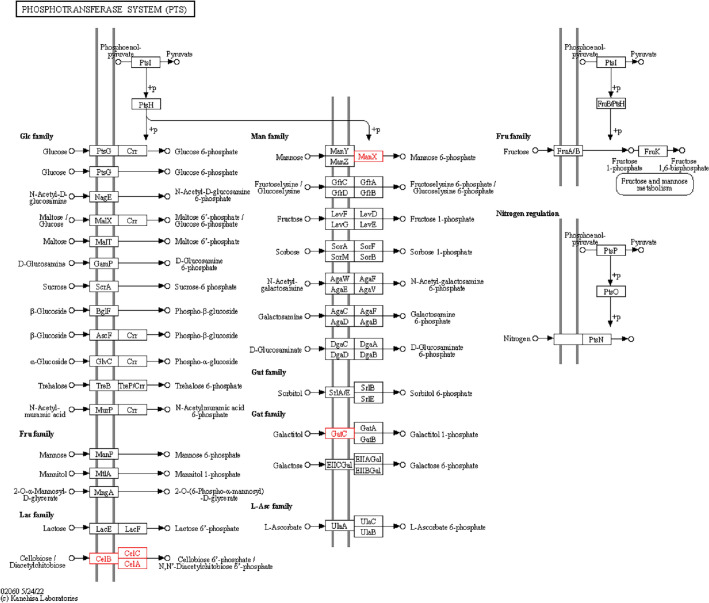
**
KO analysis of metabolic pathways.** PTS pathway, LIΔ*ilo*::*hly* vs. LI. Boxes represent proteins. Red nodes represent up-regulated proteins.

Compared with LI, the proteins expression levels of CelA, CelB, CelC, ManX, and GatC were up-regulated in LIΔ*ilo*::*hly* (Figure 6).

The quorum sensing and PTS pathway-related genes were verified using RT-qPCR (Additional files 4A–B). These results were consistent with the transcriptomic sequencing results.

### Phenotypic analysis of differential pathways

#### Biofilms

**Figure 7:**
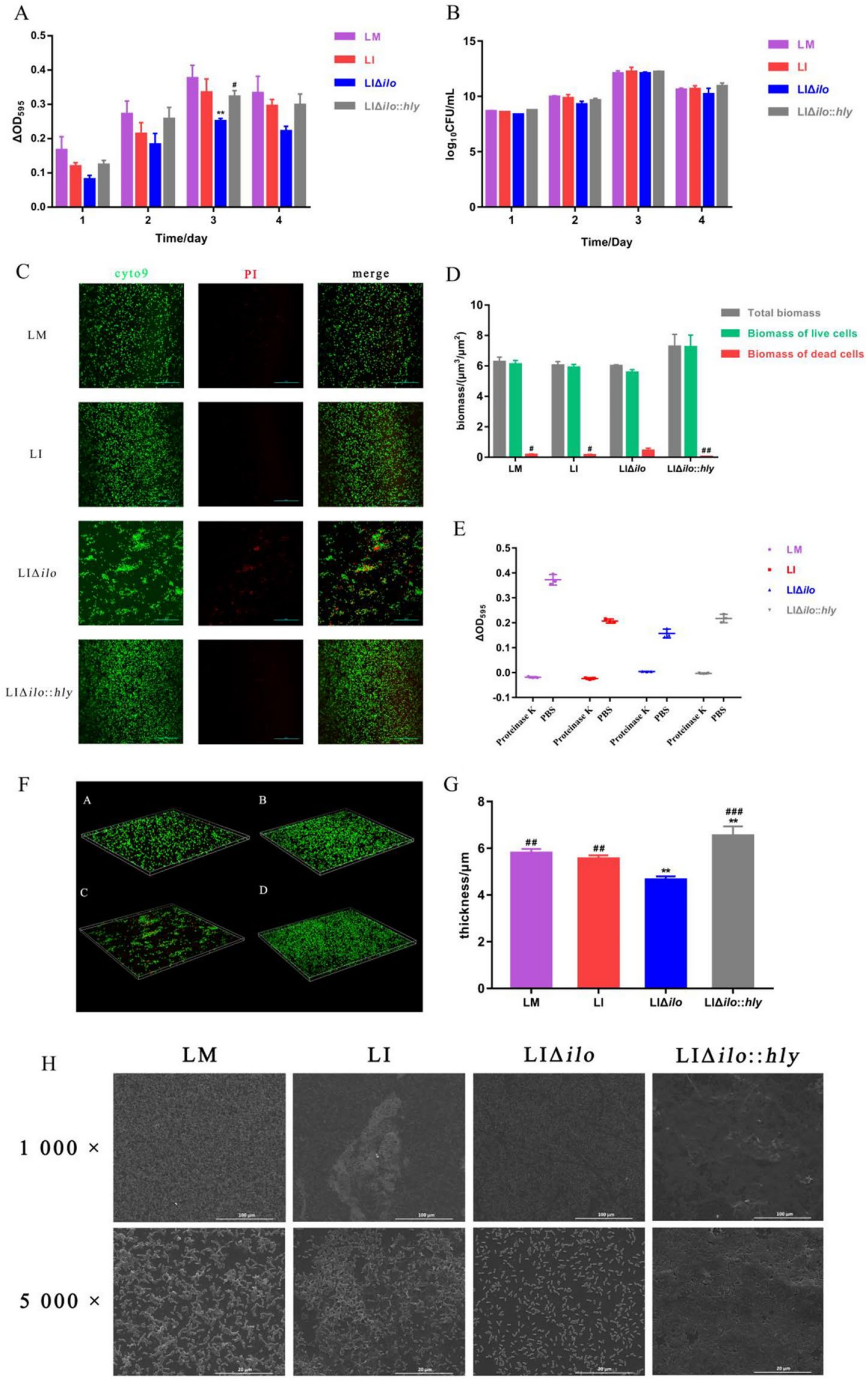
**
Biofilm formation ability.** The biofilm formation ability of each strain at different time points determined by crystal violet staining **A**; The biofilm formation ability of each strain at different time points determined by viable count method **B**; The biofilm formation ability of each strain observed by LSCM **C**; Live bacteria are labeled with cyto9 dye, dead bacteria are labeled with PI dye, and merge means the result of mixing both fluorescences. The biofilm volume of each strain observed by LSCM **D**; The biofilm residual amount of each strain after proteinase K treatment **E**; The 3D construction map of biofilm of each strain observed by LSCM **F** (a: LM, b: LI, c: LIΔ*ilo*, d: LIΔ*ilo*::*hly*); The biofilm thickness of each strain observed by LSCM **G**; Micro-images of each strains’ biofilm on the 3rd day on glass slides taken by SEM. The micro-images were taken at ×1000, ×5000 magnifications **H**; ^**^ *P* < 0.01, vs. LI; ^#^ *P* < 0.05, ^##^ *P* < 0.01, ^###^ *P* < 0.001, vs. LIΔ*ilo*; *n* = 3.

The semi-quantitative crystal violet staining method (Figure 7A) and the viable count method (Figure 7B) were used to determine the biofilm formation ability of each strain at different time points. The growth of the biofilm of the four strains peaked on the 3rd day. According to the semi-quantitative crystal violet staining, the biofilm formation ability of the hemolysin gene deletion strain was lower than that of the wild-type strain, and the ability of the modified strain to recover to the level of the wild-type strain. But there was no difference in the number of live bacteria encased in biofilm. This suggested that the difference in biofilms was not caused by the number of live bacteria, but originated from the other biomolecules that make up the biofilm, such as DNA, RNA, peptidoglycan, exopolysaccharides, proteins, and phospholipids. The biofilm formation ability of each strain was observed by laser scanning confocal microscopy (LSCM) (Figure 7C). The deletion strain was sparsely distributed, with many dead bacteria observed. The biofilm volume of each strain was determined by LSCM (Figure 7D). The total biofilm volume and viable bacterial volume of LIΔ*ilo*::*hly* group were higher than those of the other three strain groups, but the differences were not statistically significant. However, the volume of dead bacteria in the deletion strain was higher than that in the other three strains. After proteinase K treatment, the biofilms of each strain were completely degraded (Figure 7E). LSCM was also used to construct a three-dimensional (3D) map of the biofilm of each strain (Figure 7F). The deletion strain was sparsely distributed and displayed many dead bacteria, whereas the wild-type and modified strains were closely distributed and had fewer dead bacteria. The biofilm thickness of each strain was determined by LSCM (Figure 7G). The biofilm thickness of the deletion strain was lower than that of the wild-type strain, while that of the modified strain was the highest among the four strains. SEM was used to observe the biofilm formation of each strain on the 3rd day (Figure 7H).

The microscopic characterization of the biofilm can be visually observed by SEM. From the images (×5000), the obviously different microstructures of biofilms formed by different strains are shown. After *ilo* knockout, fewer bacterial cells and less exopolysaccharides were accumulated on glass slides, and after replenishing hemolysin, the strain formed a dense biofilm.

#### Virulence factor gene transcription levels

**Figure 8:**
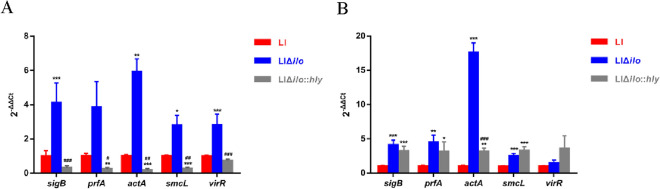
**Virulence gene expression.** The transcription levels of virulence genes in different strains when glucose **A** and cellobiose **B** were used as single carbon resource, ^*^ *P* < 0.05, ^**^ *P* < 0.01, ^***^ *P* < 0.001, vs. LI; ^#^ *P* < 0.05, ^##^ *P* < 0.01, ^###^ *P* < 0.001, vs. LIΔ*ilo*, *n =* 3.

Bacteria were cultured with a monosaccharide (glucose) and disaccharide (cellobiose) as single carbon sources. In the presence of glucose, compared with LI, the transcription levels of virulence genes of the deletion strain, except for *prfA*, were significantly up-regulated with statistical difference. After *hly* supplementation, the transcription levels of virulence genes were down-regulated more than those in LI (Figure 8A). In the presence of cellobiose, the transcription levels of virulence genes, except for *virR*, were significantly up-regulated compared with LI. Except for *smcL* and *virR*, the transcription levels of virulence genes were down-regulated after *hly* supplementation to a certain extent, but were still higher than those in LI (Figure 8B).

## Discussion

This study revealed the functional differences between LLO and ILO by predicting the basic properties and structures of LLO and ILO, and by comparing the transcriptomic analysis results of LI, LIΔ*ilo*, and LIΔ*ilo*::*hly*, which may compensate for the gaps and lack of data concerning ILO function.

The ProtParam and Compute pI/Mw online software packages were used to calculate pI. The pI of LLO (pH = 7.63) was weakly alkaline, whereas that of ILO (pH = 5.93) was weakly acidic. The pH of the lysosomal cavity in macrophages is acidic (pH = 4.5–5.0). The pI of LLO is much higher than the pH of the lysosomal cavity, while the pI of ILO is nearly equal to the pH of the lysosomal cavity. According to reports, LLO exhibits very weak cytolytic activity at neutral pH but strong activity at pH < 6. In other words, LLO exhibits weak membrane perforation activity in an environment close to its pI [21]. This is due to the presence of a pH sensor in the transmembrane structural domain of LLO. There are no definitive studies showing that ILO has similar component. Even if there is, in an environment where the pH is nearly to the pI, the effect of such a pH sensor is limited. Besides, LLO dissociates more cations in the lysosome than ILO. We speculate that these dissociated cations also have a certain proton sponge effect in the lysosome. Cationic materials have been shown to have a proton sponge effect [22]. Notably different pI may be one reason why the ability of ILO to help the bacteria escape lysosome was weaker than that of LLO. TMHMM software prediction showed that both LLO and ILO did not have a transmembrane structure and are not transmembrane proteins. Rather, they are secreted proteins. A review by Churchill et al. [23] also indicated that LLO is a secreted protein required for LM to enter the cytoplasm of host cells.

**Figure 9:**
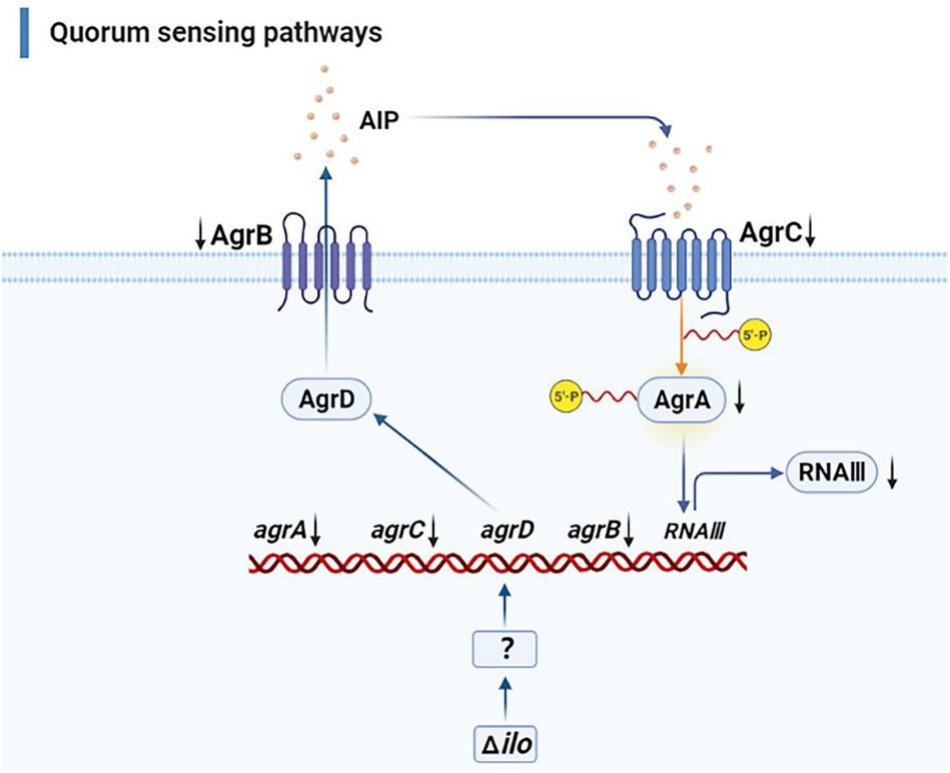
**Regulation map of quorum sensing pathway after ilo knockout (Created in BioRender.com).** AgrD precursor peptides are processed by AgrB, and autoinducing peptides (AIP) are exported. AIP releases signals through the AgrC receptor and the downstream transcription factor AgrA. Phosphorylated AgrA induces the production of regulatory factor RNA III, which controls bacterial quorum-sensing behavior and modulates virulence changes.

Transcriptome sequencing revealed that knockout of *ilo* and restoration of *hly* affected the ribosome, quorum, and PTS pathways. Among them, the quorum sensing pathway is one of the most interesting pathways, and biofilm formation is one of the most important phenotypes in the quorum sensing pathway. Biofilms are communities of microorganisms that grow on surfaces [24]. Biofilm formation includes five stages: initial bacterial colonization, extracellular matrix secretion, early formation, mature separation, and diffusion [25]. Biofilm formation is not a simple and uniform process, but rather is continuous and dynamic, and is regulated and controlled by the bacterial quorum sensing system [26]. The role of the quorum sensing system of gram-positive bacteria in regulating and controlling biofilm formation was first described in *Staphylococcus aureus*. The quorum sensing system relies on the accessory gene regulator (Agr) system [27], which consists of a quorum sensing module and a two-component system [28]. Deletion of *agrA* impairs the early biofilm formation of LM [29], and deletion of *agrD* reduces biofilm formation of LM [30]. Factors in the Agr system are important for bacteria adhesion, immune escape, and production of toxin and invasion-related protease [31]. In the present study, compared with LI, after *ilo* knockout *agrA*, *agrB*, and *agrC* were all down-regulated, and the biofilm formation ability of LIΔ*ilo* decreased. However, after restoration of *hly*, *agrB* was up-regulated to a level comparable to that of LI. The biofilm formation ability of LIΔ*ilo*::*hly* was even stronger than that of LI. Interestingly, the expression level of *agrD* did not change in this study, indicating that *ilo* knockout and complementation with *hly* may have no effect on the expression level of *agrD*. The predicted regulatory mechanism of *ilo* knockout in the quorum sensing pathway is clarified in Figure 9.

To the best of our knowledge, this is the first report regarding the direct correlation between *Listeria* hemolysin and biofilm formation.

The PTS pathway, which comprises enzyme I (EI), histidine phosphate carrier protein (HPr/NPr), enzyme II (EII) complexes, and other components [32], is present in various bacteria. This pathway is primarily involved in sugar transportation and phosphorylation. In LM, PTS affects the virulence gene regulator, PrfA, by affecting the bacterial utilization of carbon resources, thereby affecting the expression of virulence genes. *Listeria* contains a variety of virulence factors with different functions, among which environmental tolerance-related virulence factors are regulated by PrfA and SigB. The adhesion-invasion-related virulence factor sphingomyelinase C (*smcL*) is located in LI’s unique pathogenicity island (LIPI-2) [16]. This virulence factor mediates the destruction of the primary phagosome membrane and promotes bacterial intracellular proliferation, and its products are related to hemolytic activity and the ability to lyse phages [33]. Intracellular infection-related virulence factors include PrfA, LLO, and actin assembly inducing protein A (ActA). Together with the zinc metalloproteinase precursor (Mpl), they form the first pathogenicity island (LIPI-1), also known as the PrfA-dependent virulence gene cluster [34]. The two-component system response regulator VirR is the second most important virulence regulator after PrfA [35]. A study [36] found that LM mutants lacking *CelC1* and *CelR*, genes related to disaccharide metabolism in the PTS pathway, or lack of *CelA* genes, exhibited low cellulosic sugar consumption. Deletion of *CelC1* or *CelR* prevented the repression of virulence genes caused by disaccharides, but this was not applicable to glucose and fructose. Transcriptomic sequencing analysis in the present study suggested that *CelA* and *CelB* were down-regulated after *ilo* knockout and up-regulated after *hly* complementation. Such changes in the transcriptional expression of these genes affect the expression of important bacterial virulence genes. Therefore, we detected the transcription levels of five virulence genes (*sigB*, *prfA*, *actA*, *smcL*, and *virR*) of each strain grown in medium with glucose or cellobiose as the single carbon source. In the presence of glucose, the expression levels of virulence genes were up-regulated after *ilo* knockout compared with LI, and were down-regulated or even lower than those of LI after restoration of *hly*. Similar results were observed with cellobiose as the single carbon resource. After *ilo* knockout, the expression levels of most of the virulence genes were up-regulated but were still higher than those of LI. Our results were not completely consistent with the experimental results of Cao et al. [36]. We observed that the downregulation of *CelA* and *CelB* after *ilo* knockout not only down-regulated the cellobiose-induced repression of virulence genes but also down-regulated glucose-induced repression. Notably, our transcriptome sequencing results did not reveal that *ilo* knockout altered the expression levels of genes involved in glucose metabolism in the PTS pathway. The specific mechanism underlying this change and whether it is mediated by the PTS pathway remain to be assessed.

Transcriptome sequencing analysis revealed that the quorum sensing and PTS pathways were significantly altered after the knockout of *ilo* and restoration of *hly*. These two pathways play an important role in regulating bacterial virulence, which is closely related to bacteria infecting the host and the host immune response to pathogens. Immune evaluations of the three strains at the cellular and animal levels must be performed. Further studies are needed to determine whether LIΔ*ilo*::*hly* can elicit more immune responses than LI and whether LIΔ*ilo*::*hly* is more effective than LI. In this study, we only examined the biofilm formation ability related to the quorum sensing pathway and the expression of virulence genes related to the PTS pathway. The results of the prokaryotic transcriptome sequencing require further investigation. In addition, only the reference strains and their derived strains were studied in this research. It was considered that the wild strain may show different biofilm formation ability and higher adaptability and persistence than the reference strain. Therefore, subsequent studies should include more wild strains. Transcriptome sequencing after infecting macrophages or mice with the strains must be performed to reveal the immunoregulatory mechanisms of ILO and LLO in vivo and the tumor microenvironment.

## Supplementary Information

**Additional file 1.** **Construction of recombinant strains.** Schematic diagram of targeting plasmids pCW619, pCW620 and pCW621 (A). Schematic diagram of the recombinant strains (B).

**Additional file 2.** **Bioinformatics analysis of LLO and ILO proteins.** Amino acid composition of LLO (A) and ILO (B); Secondary structures of LLO (C) and ILO (D) predicted by Predict Protein software; Secondary structures of LLO (E) and ILO (F) predicted by SOPMA software (*α*-helix in blue, extended strand in red, *β*-turnin green, random coil in orange); Hydrophilic and hydrophobic prediction of LLO (G) and ILO (H); Transmembrane structure prediction of LLO (I) and ILO (J); Functional domain prediction of LLO (K) and ILO (L).

**Additional file 3.** **KO analysis of metabolic pathways.** Quorum sensing pathway, LIΔ*ilo* vs. LI (A), LIΔ*ilo*::*hly* vs. LIΔ*ilo* (B), LIΔ*ilo*::*hly* vs. LI (C). The figure shows a portion of the quorum sensing pathway, which is related to the regulation of biofilms. Boxes generally represent proteins. Red nodes represent proteins whose expression levels are up-regulated. Green nodes represent down-regulated proteins.

**Additional file 4.** **Validation of differentially expressed genes by RT-qPCR.** RT-qPCR results of quorum sensing pathway-related genes and other genes (A), RT-qPCR results of PTS pathway-related genes (B), 10410, 10415: SIS domain-containing protein; 03675: beta-glucoside-specific PTS transporter subunit IIABC, 11845: fructose-specific PTS transporter subunit EIIC; 08975, 13725: PTS sugar transporter subunit IIB; 13730, 14020: PTS sugar transporter subunit IIC.

## Data Availability

The datasets used and/or analysed during the current study are available from the corresponding author on reasonable request.
