# Supplementary figures and images for: Hemolysin function of Listeria is related to biofilm formation: transcriptomics analysis
Source: Vet Res. 2022 Dec 31;53:113. doi: 10.1186/s13567-022-01124-y (PMC9805692; doi:10.1186/s13567-022-01124-y)

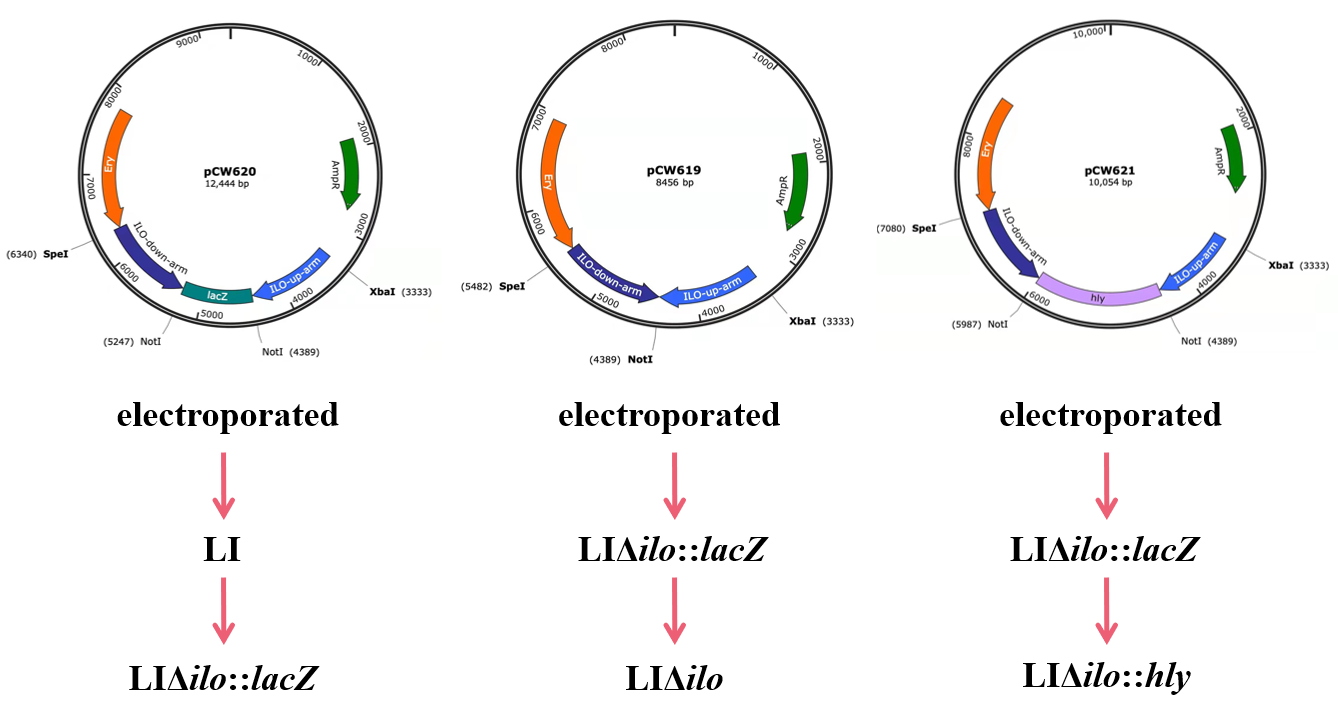


A


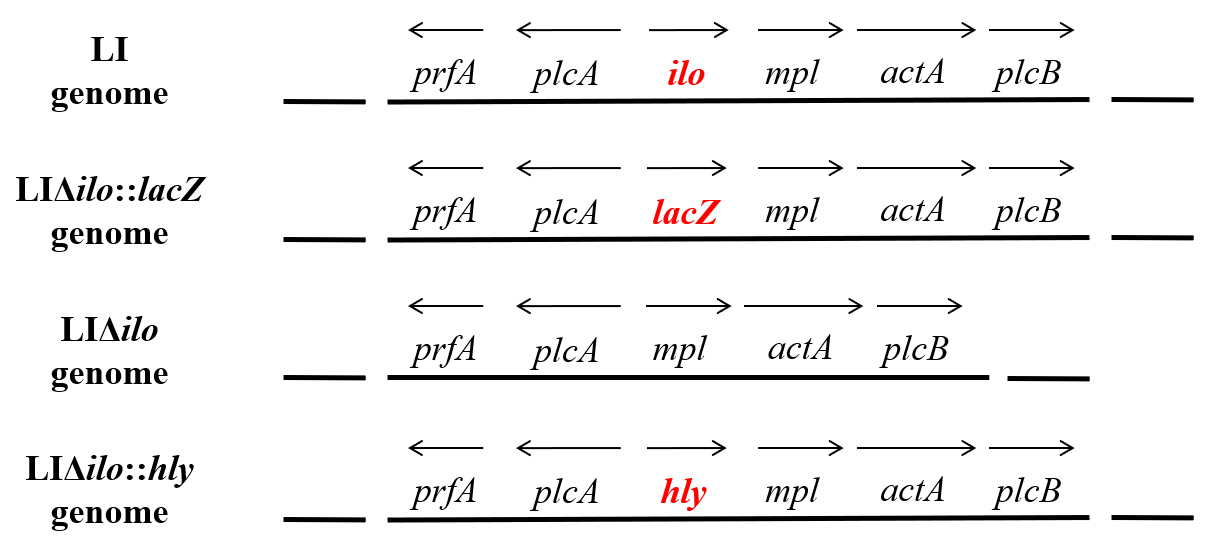


B

Supplement: Supplementary file 1 — Additional file 1. Construction of recombinant strains. Schematic diagram of targeting plasmids pCW619, pCW620 and pCW621 (A). Schematic diagram of the recombinant strains (B). [file 13567_2022_1124_MOESM1_ESM.docx]

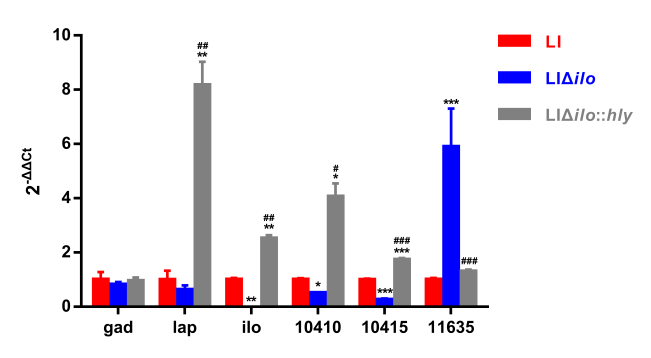


A

B


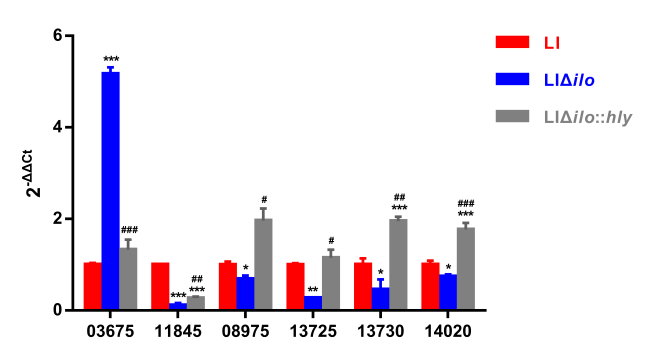

Supplement: Supplementary file 4 — Additional file 4. Validation of differentially expressed genes by RT-qPCR. RT-qPCR results of quorum sensing pathway-related genes and other genes (A), RT-qPCR results of PTS pathway-related genes (B), 10410, 10415: SIS domain-containing protein; 03675: beta-glucoside-specific PTS transporter subunit IIABC, 11845: fructose-specific PTS transporter subunit EIIC; 08975, 13725: PTS sugar transporter subunit IIB; 13730, 14020: PTS sugar transporter subunit IIC. [file 13567_2022_1124_MOESM4_ESM.docx]
