# Supplementary material for: Hemolysin function of Listeria is related to biofilm formation: transcriptomics analysis
Source: Vet Res. 2022 Dec 31;53:113. doi: 10.1186/s13567-022-01124-y (PMC9805692; doi:10.1186/s13567-022-01124-y)
Supplement: Supplementary file 2 [file 13567_2022_1124_MOESM2_ESM.docx]

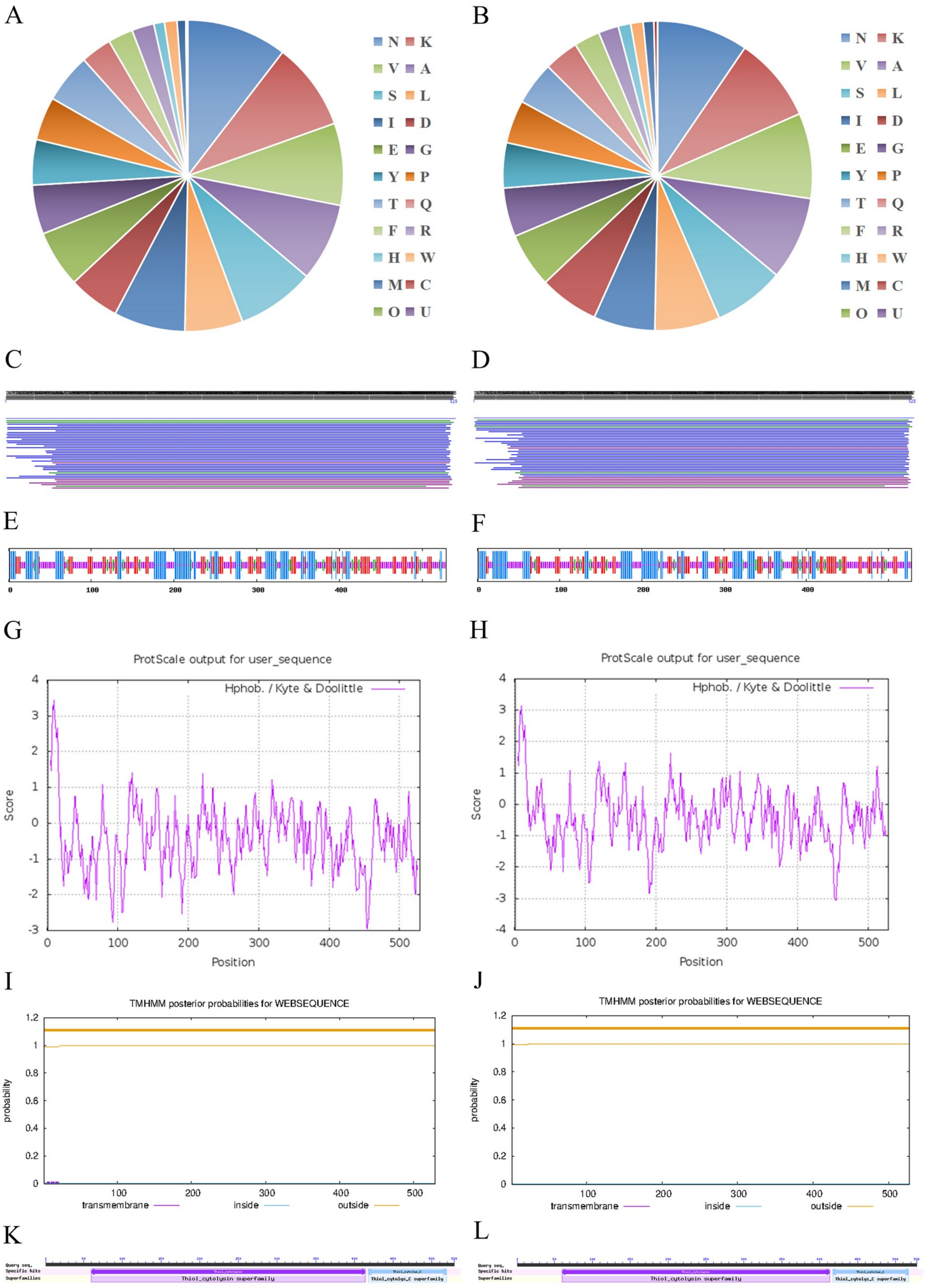

A
B
N K
V A
S L
I D
E G
Y P
T Q
F R
H W
M C
O U
C
D
E
F
0
100
200
300
400
G
H
ProtScale output for user_sequence
Hphob. / Kyte & Doolittle
Score
Position
I
J
TMHMM posterior probabilities for WEBSEQUENCE
probability
transmembrane
inside
outside
K
L
Query seq.
Specific hits
Superfamilies
Thiol_cytolysin superfamily
Thiol_cytolys_C superfamily
