# Supplementary material for: Hemolysin function of Listeria is related to biofilm formation: transcriptomics analysis
Source: Vet Res. 2022 Dec 31;53:113. doi: 10.1186/s13567-022-01124-y (PMC9805692; doi:10.1186/s13567-022-01124-y)
Supplement: Supplementary file 3 [file 13567_2022_1124_MOESM3_ESM.docx]

A

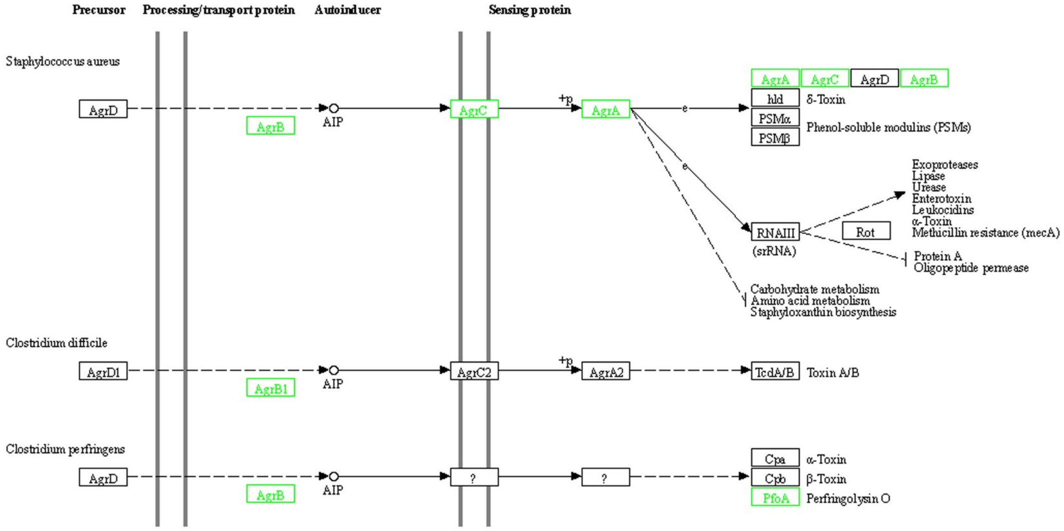
Precursor
Processing/transport protein
Autoinducer
Sensing protein
Staphylococcus aureus
AgrD
AgrB
AIP
AgrC
+p
AgrA
AgrA
AgrC
AgrD
AgrB
hld
δ-Toxin
PSMα
PSMβ
Phenol-soluble modulins (PSMs)
RNAIII
(srRNA)
Rot
Exoproteases
Lipase
Urease
Enterotoxin
Leukocidins
α-Toxin
Methicillin resistance (mecA)
Protein A
Oligopeptide permease
Carbohydrate metabolism
Amino acid metabolism
Staphyloxanthin biosynthesis
Clostridium difficile
AgrD1
AgrB1
AIP
AgrC2
+p
AgrA2
TcdA/B
Toxin A/B
Clostridium perfringens
AgrD
AgrB
AIP
?
?
Cpa
α-Toxin
Cpb
β-Toxin
PfoA
Perfringolysin O

B

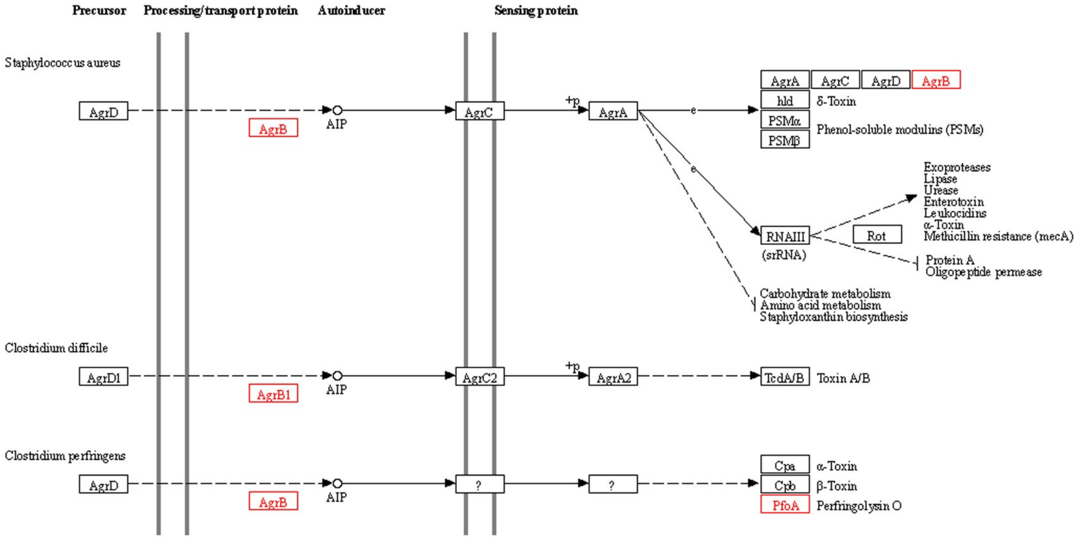
Precursor
Processing/transport protein
Autoinducer
Sensing protein
Staphylococcus aureus
AgrD
AgrB
AIP
AgrC
+p
AgrA
AgrA
AgrC
AgrD
AgrB
hld
δ-Toxin
PSMα
PSMβ
Phenol-soluble modulins (PSMs)
RNAIII
(srRNA)
Rot
Exoproteases
Lipase
Urease
Enterotoxin
Leukocidins
α-Toxin
Methicillin resistance (mecA)
Protein A
Oligopeptide permease
Carbohydrate metabolism
Amino acid metabolism
Staphyloxanthin biosynthesis
Clostridium difficile
AgrD1
AgrB1
AIP
AgrC2
+p
AgrA2
TcdA/B
Toxin A/B
Clostridium perfringens
AgrD
AgrB
AIP
?
?
Cpa
α-Toxin
Cpb
β-Toxin
PfoA
Perfringolysin O

C

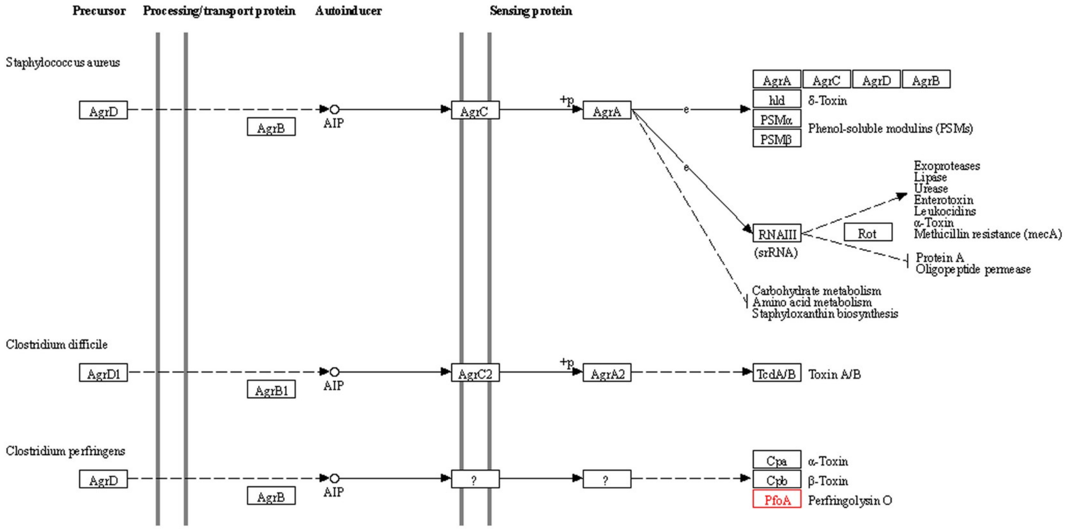
Precursor
Processing/transport protein
Autoinducer
Sensing protein
Staphylococcus aureus
AgrD
AgrB
AIP
AgrC
+p
AgrA
AgrA
AgrC
AgrD
AgrB
hld
δ-Toxin
PSMα
PSMβ
Phenol-soluble modulins (PSMs)
RNAIII
(srRNA)
Rot
Exoproteases
Lipase
Urease
Enterotoxin
Leukocidins
α-Toxin
Methicillin resistance (mecA)
Protein A
Oligopeptide permease
Carbohydrate metabolism
Amino acid metabolism
Staphyloxanthin biosynthesis
Clostridium difficile
AgrD1
AgrB1
AIP
AgrC2
+p
AgrA2
TcdA/B
Toxin A/B
Clostridium perfringens
AgrD
AgrB
AIP
?
?
Cpa
α-Toxin
Cpb
β-Toxin
PfoA
Perfringolysin O
